# A novel WST-8-based colorimetric method for sensitive and interference-free determination of glutathione peroxidase activity

**DOI:** 10.1093/biomethods/bpag049

**Published:** 2026-08-27

**Authors:** Ayat A Abdulhassan, Mahmoud Hussein Hadwan

**Affiliations:** Department of Chemistry, College of Science, University of Babylon, Hillah, Babylon Governorate 51002, Iraq; Department of Chemistry, College of Science, University of Babylon, Hillah, Babylon Governorate 51002, Iraq

**Keywords:** antioxidant enzymes, WST-8, oxidative stress, colorimetric assay, enzymatic activity, biochemical diagnostics

## Abstract

Glutathione peroxidase (GPx) is a major antioxidant enzyme that protects cells from oxidative damage, and accurate quantification of GPx is important for biochemical, clinical, and diagnostic research. Current colorimetric and spectrophotometric GPx assays, however, are often limited by low sensitivity, unstable reagents, or interference from biological matrix components. We developed a colorimetric GPx assay based on the tetrazolium salt WST-8. GPx reduces peroxide substrates (ROOH) to water or the corresponding alcohols using reduced glutathione (GSH) as an electron donor, generating oxidized glutathione (glutathione disulfide, GSSG); glutathione reductase (GR) then regenerates GSH from GSSG at the expense of NADPH. Unreacted NADPH reduces WST-8 to a stable, water-soluble formazan dye measured at 460 nm, so GPx activity can be calculated indirectly from the extent of NADPH consumption. Assay performance was compared with established DTNB- and UV-based GPx methods. The WST-8-based assay showed high sensitivity, a wide linear range, good reagent stability, and strong resistance to interference from sugars, amino acids, and proteins. Activity values obtained with the new method correlated closely with those from the conventional DTNB assay, confirming its accuracy. The WST-8-GPx assay offers improved sensitivity and interference resistance while remaining operationally simple and compatible with routine microplate instrumentation, making it a practical option for both clinical diagnostics and oxidative stress research.

## Graphical abstract

**Graphical Abstract:**
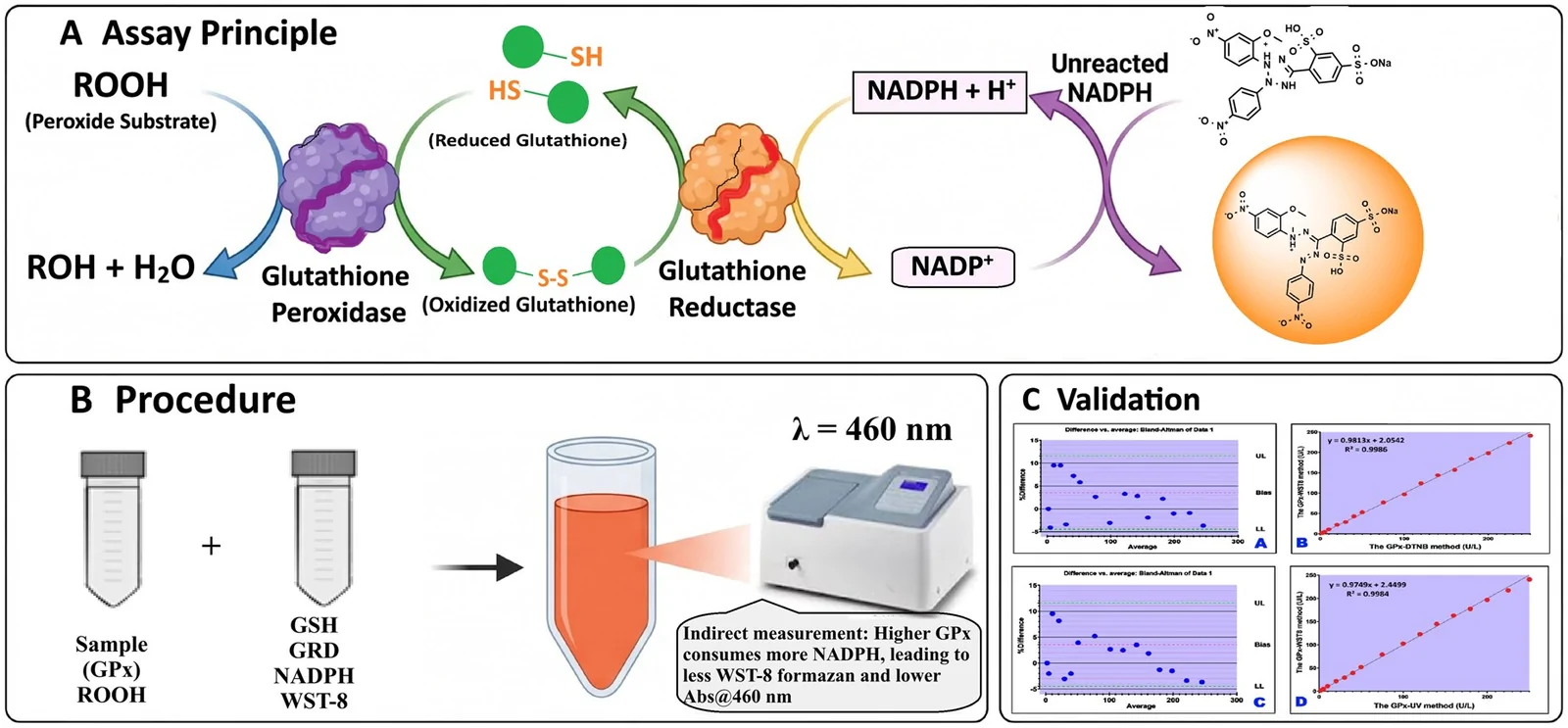

## Background

Glutathione peroxidase (GPx) is a selenoenzyme first identified by Mills in mammalian erythrocytes in 1957 [1]. Because each subunit contains a catalytic selenium atom, GPx is considered one of the principal enzymes protecting cells from oxidative damage, with particularly high activity in endothelial cells of the lung. Within the cell, approximately 60%–75% of GPx activity is localized to the cytoplasm and 25%–40% to the mitochondria, with the highest overall activity observed in erythrocytes and liver [2].

Mechanistically, GPx detoxifies hydrogen peroxide (H_2_O_2_) and lipid peroxides using reduced glutathione (GSH) as the electron donor, thereby oxidizing GSH to glutathione disulfide (GSSG) [3]. Together with catalase, GPx provides one of the two principal routes for cellular H_2_O_2_ detoxification [4]; through this activity, it protects membrane lipids and hemoglobin from peroxidative damage and contributes to xenobiotic detoxification, acting alongside catalase and superoxide dismutase as part of a coordinated antioxidant defense system [5].

This role makes GPx, particularly its cytosolic form, essential for maintaining cellular structure and function as the primary defense against lipid peroxidation [6]. A related selenoprotein, phospholipid hydroperoxide GPx (PHGPx), is a monomeric cytosolic enzyme that directly reduces membrane phospholipid hydroperoxides to their corresponding alcohols, providing a second line of defense against membrane peroxidation when the membrane-bound antioxidant vitamin E is insufficient [7, 8].

More broadly, the GPx family comprises heme-free thiol peroxidases and selenoenzymes that reduce H_2_O_2_ and organic hydroperoxides to water or their corresponding alcohols [9]. Because catalytic activity depends on selenocysteine (Sec), enzyme activity is closely tied to the body’s selenium status [10]. Eight GPX isoforms are known in mammals (GPx1–GPx8); GPx1–GPx4 and GPX6 are selenocysteine-containing selenoproteins with antioxidant function, and GPx6 is notably expressed only in humans [11, 12].

Given this broad physiological importance, reliable measurement of GPx activity remains essential for both research and clinical practice. Established methods generally fall into two categories: those that monitor substrate consumption, GSH depletion, or hydroperoxide utilization at defined time points, and those that use an indirect coupled-enzyme approach, linking GPx activity to a glutathione reductase (GR)–catalyzed reaction that allows continuous monitoring of NADPH consumption as a surrogate for GSSG production [13].

The most widely used substrate-consumption method employs Ellman’s reagent (5,5′-dithiobis-(2-nitrobenzoic acid), DTNB) to colorimetrically measure GSH consumption [14, 15]. Although broadly applicable, the DTNB assay is limited by relatively low sensitivity, reagent instability, and the need for acid-mediated reaction termination and protein precipitation, which complicate adaptation to high-throughput microplate formats.

The coupled GR–NADPH method introduced by Paglia and Valentine [16] is the most widely used indirect assay, monitoring the decay of NADPH absorbance at 340 nm. Although it facilitates continuous kinetic measurement with acceptable selectivity, it has the disadvantage that strong ultraviolet absorption by proteins and nucleic acids at 340 nm diminishes sensitivity in tissue samples. Additionally, the GR and NADPH reagents are comparatively expensive [17].

Alternative approaches include the CUPRAC method, which is based on the reduction of a copper(II)–neocuproine complex by unreacted GSH, with detection at 450 nm [18]. Although adaptable to microplate formats, this method is susceptible to interference from endogenous catalase, which competes with GPx for H_2_O_2_. A later modification removed the catalase-termination step to improve performance in complex biological matrices, though sensitivity to matrix components remains a concern [19]. Fluorometric assays using reagents such as *o*-phthalaldehyde offer high sensitivity and require only small sample volumes, but they require derivatization steps and specialized instrumentation that limit routine clinical use [20]. More recently, a Tiron-based protocol using ferrous ions for reaction termination and detection at 570 nm has shown high accuracy, precision, and minimal interference [21], although it requires freshly prepared reagents and strict pH control and has not yet achieved widespread adoption.

Collectively, despite decades of methodological development, available GPx activity assays continue to face challenges related to biological matrix interference, reagent cost, and access to detection instrumentation, particularly in resource-limited laboratory settings. There therefore remains a clear need for a sensitive, cost-effective, and interference-resistant method suited to reliable GPx quantification in complex biological samples.

Tetrazolium salts are a versatile class of chromogenic compounds that have been widely used in biochemistry, cell biology, and microbiology for over half a century [22]. Their utility arises from the reduction of the tetrazole ring by intracellular reductants, primarily NADH and NAD(P)H, to yield intensely colored, water-soluble formazan products that are readily quantified by absorbance spectrophotometry [23]. Among these, WST-8 (2-[2-methoxy-4-nitrophenyl]-3-[4-nitrophenyl]-5-[2,4-disulfophenyl]-2H-tetrazolium sodium salt), also known as CCK-8, is a cell-impermeable tetrazolium salt that forms a stable, water-soluble formazan complex upon reaction with NADPH, measurable at 460 nm [24, 25]. These properties make WST-8 a particularly attractive probe for enzymatic assays requiring sensitive, real-time detection of NAD(P)H consumption.

The present study introduces a novel WST-8-based colorimetric method for the quantitative determination of GPx activity. The assay exploits a coupled enzymatic strategy in which GPx reduces peroxide substrates (ROOH) using GSH to produce GSSG, which is then recycled to GSH by GR using NADPH; the residual, unreacted NADPH reduces WST-8 to a chromogenic complex with an absorbance maximum at 460 nm, allowing GPx activity to be quantified indirectly from NADPH consumption. This approach offers a sensitive, operationally straightforward, and interference-resistant alternative to existing methods, with broad potential application in both laboratory research and clinical diagnostic settings.

## Procedure

### Chemicals and reagents

H_2_O_2_ (30%), sodium azide, sodium hydroxide, sodium phosphate dibasic (Na_2_HPO_4_), potassium phosphate monobasic (KH_2_PO_4_), hydrochloric acid, sulfuric acid, ethanol, Triton X-100, cysteine, and ascorbic acid were purchased from Thomas Baker (Chemicals) Pvt. Ltd.

Bovine serum albumin, reduced glutathione, Ellman’s reagent (DTNB), cumene hydroperoxide (CuOOH), *tert*-butyl hydroperoxide (tBuOOH, 70 wt% in H_2_O), β-nicotinamide adenine dinucleotide 2′-phosphate reduced tetrasodium salt hydrate (NADPH), and GR were purchased from Sigma-Aldrich. Phosphatidylcholine and cholesterol were obtained from Sigma.

WST-8/1-methoxy-5-methylphenazinium methylsulfate (1-mPMS) was purchased from Elabscience.

### Preparation of buffers and stock solutions

#### Phosphate buffer (100 mM, pH 7.2)

Solution A was prepared by dissolving 13.62 g KH_2_PO_4_ in 1 l distilled water, and solution B by dissolving 17.8 g Na_2_HPO_4_·2H_2_O in 1 l distilled water. Equal volumes of solutions A and B were combined, and sodium azide (0.372 g) and EDTA (0.6501 g) were added as preservative and chelating agent, respectively.

#### Reduced glutathione (GSH) solutions

A 10 mM GSH stock for the GPx-WST8 and GPx-UV assays was prepared fresh on each day of use: solid GSH (MW 307.3) was equilibrated to room temperature for 30 minutes, weighed at 3.1 g/l (scaled to the volume needed), and dissolved in assay buffer (100 mM sodium phosphate, pH 7.2); fresh preparation was necessary because GSH is prone to oxidation, and day-old stocks gave inconsistent results. For the GPx-DTNB reference assay, a separate 2 mM GSH solution was prepared by dissolving 0.1228 g reduced glutathione in 100 ml of the 100 mM phosphate buffer (pH 7.2).

#### β-NADPH solution (2 mM, fresh)

Dry β-NADPH was equilibrated to room temperature for 30 minutes, weighed at 1.5 g/l (scaled to the required volume), and dissolved in room temperature assay buffer; as with GSH, this solution was prepared fresh each assay day to avoid oxidative loss of activity.

#### H_2_O_2_  *solutions*

For the GPx-WST8 and GPx-UV assays, a 5 mM H_2_O_2_ working solution was prepared by diluting 6 µl of concentrated (30%) H_2_O_2_ in 10 ml of water; the exact concentration was verified using the molar extinction coefficient of H_2_O_2_ (43.6 M^−1^ cm^−1^ at 240 nm). The diluted solution was stored at 4°C in an iron-free container, remained stable for several days, and was periodically checked by monitoring the blank assay rate. For the GPx-DTNB reference assay, a 2 mM H_2_O_2_ solution was prepared daily in 100 mM phosphate buffer (pH 7.2), with concentration verified using the molar extinction coefficient of H_2_O_2_ (43.6 M^−1^cm^−1^ at 240 nm).

#### GR solution (100 U/ml)

The ammonium sulfate suspension of GR was diluted in a small volume of ice-cold assay buffer to a final activity of 100 U/ml, kept on ice, and used within 2–3 days, as activity declined with longer storage at 4°C.

#### Cumene hydroperoxide working stock

Commercial cumene hydroperoxide (7.8 M) was first diluted in ethanol to prepare an intermediate 5× stock (0.15 M), then diluted five-fold in water to yield a 20× working stock (30 mM); 50 µl of this stock added to a 1-ml cuvette gave a final reaction concentration of 1.5 mM. Where a 5 mM working solution was required, it was obtained by diluting the 30 mM stock six-fold in water (one part stock + five parts buffer). Cumene hydroperoxide has a pungent odor and is hazardous; the concentrated stock was handled in a fume hood, with gloves worn throughout.

#### tert-Butyl hydroperoxide (tBuOOH) solution

A 1 M stock was prepared by adding 130 µl of 70% tBuOOH (∼7.7 M) to 870 µl of 100 mM phosphate buffer (pH 7.2); a 2 mM working solution was then prepared by diluting 200 µl of the 1 M stock into 99.8 ml of phosphate buffer.

#### WST-8/1-mPMS detection reagent

The commercial WST-8/1-mPMS reagent (Elabscience), containing 0.2 mM 1-mPMS and 5 mM WST-8, was stored at –20°C and used as supplied.

### Protein concentration determination

Protein concentration was determined using the Bradford Protein Colorimetric Assay Kit (Elabscience; Cat. No. E-BC-K168-M).

## Biological samples

### Blood specimens

Three milliliters of whole blood were collected into heparinized tubes for preparation of erythrocyte lysates. Tubes were centrifuged at 400 × g for 10 minutes to separate plasma and buffy coat, and red blood cells were washed 3× with 500 µl of 0.9% NaCl. An aliquot (500 µl) of the erythrocyte suspension was then combined with 2 ml of ice-cold double-distilled water, vortexed for 10 seconds, and kept in the dark at 4°C for 15 min. The resulting hemolysate was diluted 1:500 in 100 mM phosphate-buffered saline (PBS) and used as the source of GPx activity.

### Tissue preparation

Male albino rats and mice were obtained from the Bioscience Department animal house at the University of Babylon (Iraq). Livers were surgically excised, rinsed with 0.9% (w/v) NaCl to remove blood and contaminants, and homogenized in cold 1.15% (w/v) KCl using a glass homogenizer. The homogenate was filtered through two layers of muslin to remove cellular debris, and the filtrate was diluted 1:500 in 100 mM PBS for subsequent GPx activity assays.

### Ethics approval

The study protocol was approved by the College of Science Ethics Committee at the University of Babylon, Iraq (Ref. No. 2263Q, dated 29 September 2025).

### GPx-WST8 assay protocol

The cuvette-based assay protocol is summarized in Table 1. Assay buffer, GSH, β-NADPH, sodium azide, and GR were combined with the study sample, and the reaction was initiated by adding peroxide. Tubes were vortexed and incubated at 37°C for 10 minutes, then placed in a boiling water bath for 3 minutes and cooled in an ice bath for 4 minutes to terminate the enzymatic reaction. WST-8 reagent (50 µl) was then added, and the tubes were vortexed and incubated at 37°C for an additional 10 minutes. Finally, 250 µl of 2 M acetic acid was added, and samples were incubated for 10 minutes at 37°C before absorbance was measured at 460 nm.

**Table 1:** Protocol for measurement of glutathione peroxidase activity.^a^

Reagent	Test	Standard	Control	Blank
Assay buffer (100 mM sodium phosphate, pH 7.2)	0.63 ml	0.78 ml	0.78 ml	0.83 ml
GSH (10 mM)	0.10 ml	0.10 ml	0.10 ml	0.10 ml
β-NADPH (2 mM)	0.10 ml	0.10 ml	—	—
Sodium azide (1.125 M)	0.01 ml	0.01 ml	0.01 ml	0.01 ml
Glutathione reductase (100 U/ml)	0.01 ml	0.01 ml	0.01 ml	0.01 ml
Study sample	0.10 ml	—	—	—
Sample background control	—	—	0.10 ml^b^	—
5 mM H_2_O_2_ (added last, to start the reaction)	0.05 ml	—	—	0.05 ml

a The test tubes were vortexed and incubated at 37°C for 10 minutes, then immersed in a boiling water bath for 2 minutes and cooled in an ice bath for 4 minutes. WST-8 (100 µl) was then added to each tube, and the mixtures were vortexed and incubated at 37°C for 10 minutes. Following this, 250 µl of 2 M CH_3_COOH was added to each tube, and the samples were incubated for an additional 10 minutes at 37°C. Absorbance was subsequently measured at 460 nm.

b Denotes the sample background control used without added substrate. Sample background control was prepared by boiling the sample for two minutes, then cooling it in an ice-water bath.

### Calculation of GPx activity

One unit of GPx activity is defined as the amount of enzyme that oxidizes 1.0 µmol of GSH to GSSG per minute at 25°C and pH 7.2; equivalently, as the amount of enzyme that oxidizes 1.0 µmol of NADPH to NADP^+^ per minute under the same conditions.


(1) Residual NADPH concentration in the test tube=[(A Test−A Control)/ A STD]×concentration of STD

**Figure 1:**
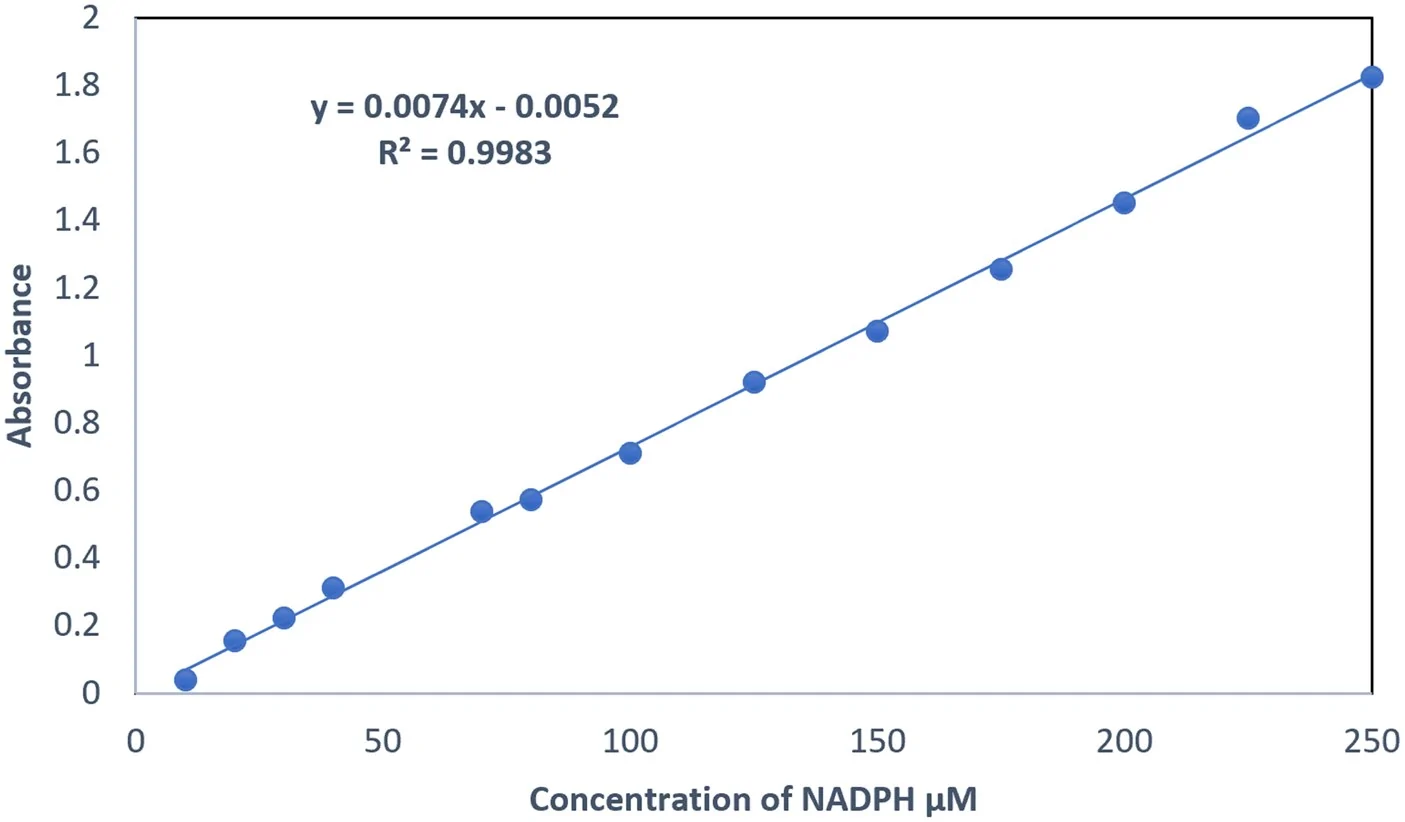
Standard curve relating absorbance (460 nm) to NADPH concentration, used to calculate residual NADPH in the GPx-WST8 assay.

The peroxide standard curve (Fig. 1) was used to determine the residual NADPH concentration in each test tube; the number of micromoles of peroxide consumed equals the GPx activity.


(2) GPx activity (U/L)={[concentration of NADPH in STD−concentration of residual NADPH in test tube]/ time (10 min)}×[total volume (mL)/ sample volume (mL)]×dilution factor

### Dilution factor

The dilution factor (DF) corrects for any sample dilution performed prior to analysis and must be accounted for when interpreting the results.

### Standard curve

The assay standard curve was constructed by diluting an NADPH standard in 0.1 M phosphate buffer (pH 7.2) to final concentrations ranging from 0.5 to 250 µM. Each standard was processed according to the protocol in Table 1, and absorbance was measured at 460 nm relative to the blank after 5 minutes. The resulting standard curve (Fig. 1) was linear across the tested range.

## Reference methods

### GPx-DTNB

GPx activity (EC 1.11.1.9) was determined as described by Sattar *et al.* [15]. The reaction medium contained 1.5 ml of phosphate buffer (0.01 M, pH 7.2), 0.2 ml of reduced glutathione (2 mM), and 0.05 ml of sample supernatant. The reaction was initiated with 0.2 ml of H_2_O_2_ (2.1 mM) and incubated for 60 minutes. Then, 3 ml of sodium hydrogen phosphate and 1 ml of Ellman’s reagent were added, and samples were held at 37°C for 10 minutes before optical density was measured at 412 nm.

### GPx-UV

GPx activity was measured using the UV method of Paglia and Valentine [16]. GPx catalyzes oxidation of 0.10 ml of GSH (10 mM) by 0.05 ml of H_2_O_2_ (5 mM); the resulting GSSG is converted back to GSH by 0.01 ml of GR (100 U/ml) in the presence of 0.1 ml of NADPH (2 mM), and the resulting decrease in absorbance was measured at 340 nm.

## Method validation

### Accuracy, selectivity, and reproducibility

Validation followed the bioanalytical method validation guidance [26]. To assess accuracy and specificity of the GPx-WST8 assay under conditions simulating physiological interference, four test groups were prepared in 10 ml of 100 mM phosphate buffer (pH 7.2): (i) a control containing only buffer; (ii) a mixture of sugars (fructose, xylose, and sucrose, 2 mM each) simulating carbohydrate interference; (iii) a mixture of amino acids (histidine, cysteine, methionine, and threonine, 2 mM each) simulating amino acid interference; and (iv) 0.5% bovine serum albumin (BSA) plus 0.5% casein simulating protein interference. GPx enzyme solution (1 ml, 250 U/l) was added to each vial, and activity was measured using the GPx Assay Kit (Catalog No. E-BC-K809-M, Elabscience, China). Recovery was calculated for each interfering condition by comparing measured activity with the expected activity in the control. Within-day and between-day precision were assessed using biological samples spiked with known GPx concentrations, expressed as relative standard deviation (RSD).

### Sensitivity and linearity

The GPx-WST8 assay was evaluated across a range of NADH concentrations (0.05 to 500 µM) to assess sensitivity and linearity. Sensitivity was characterized by the limits of quantification (LOQ) and detection (LOD).

### Statistical validation

The GPx-WST8 method was compared with the GPx-DTNB and GPx-UV reference methods using GraphPad Prism 8.0 to estimate bias and compare analytical performance. Bland–Altman analysis was used to determine limits of agreement among the GPx-WST8, GPx-DTNB, and GPx-UV methods, plotting the absolute difference between paired measurements against their mean; per Bland and Altman, the 95% limits of agreement correspond to the mean difference ± 1.96 standard deviations. Passing–Bablok regression was additionally used to assess agreement between methods and to identify systematic bias. The comparison samples were generated from a single pooled sample series, serially diluted with phosphate buffer to obtain 16 distinct GPx activity levels spanning the assay’s analytical range (2–250 U/l). Each of the 16 dilution levels was assayed in triplicate (*n* = 3) by all three methods (GPx-WST8, GPx-DTNB, GPx-UV) on the same day, yielding 48 paired data points per method comparison (16 concentration levels × 3 replicates) that were used to perform the Bland–Altman and Passing–Bablok analyses.

## Results

### Principle of the GPx-WST8 assay

**Figure 2:**
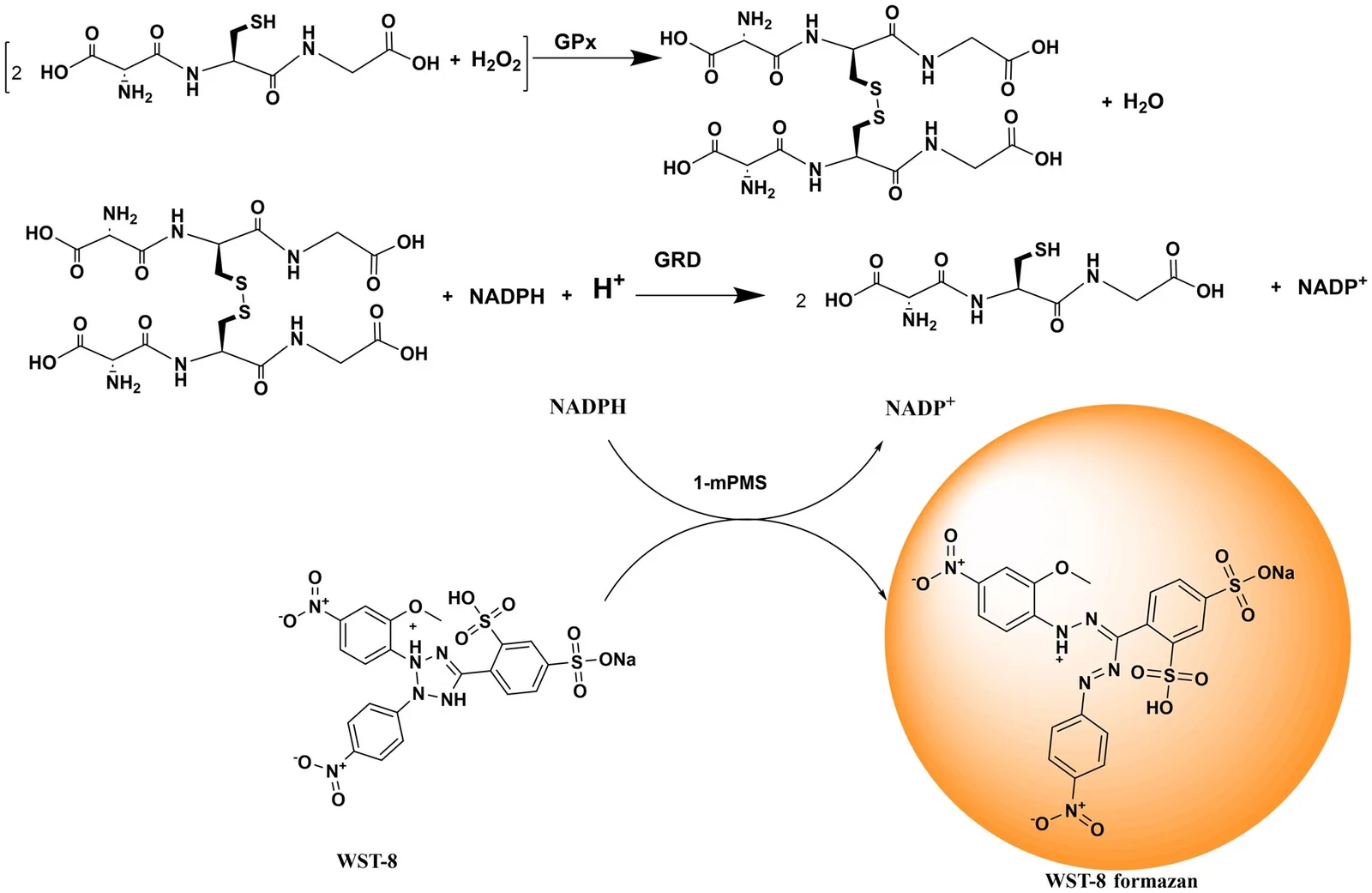
GPx reduces peroxide substrates (ROOH) to water or the corresponding alcohols (ROH) using reduced glutathione (GSH), generating oxidized glutathione (GSSG); glutathione reductase then regenerates GSH from GSSG at the expense of NADPH. Unreacted NADPH reduces WST-8 to a stable chromogenic formazan product.

In the modified endpoint assay, GPx reduces a peroxide substrate (ROOH) to water or the corresponding alcohol (ROH) using GSH as the electron donor, generating oxidized glutathione (GSSG); GR then regenerates GSH from GSSG at the expense of NADPH (as shown in Fig. 2). Unreacted NADPH remaining at the end of the reaction reduces WST-8 to a stable, water-soluble formazan product with an absorbance maximum at 460 nm. Because the amount of unreacted NADPH is inversely related to GPx activity, the intensity of the formazan color and the corresponding absorbance at 460 nm are likewise inversely proportional to GPx activity in the sample. One international unit (IU) of GPx activity is defined as the amount of enzyme required to consume 1 µmol of NADPH per minute at 37°C.

WST-8 is not reduced directly and efficiently by NADPH: the redox potential and kinetics of a direct hydride transfer from NADPH to the tetrazolium ring of WST-8 are unfavorable, and this reaction alone would be too slow to support a reproducible endpoint assay within a practical incubation time [24, 25]. The 1-methoxy-5-methylphenazinium methylsulfate (1-mPMS) functions as a low molecular weight electron transfer mediator that bridges this gap, in the same manner it is employed in established NAD(P)H-detection and cell viability formats such as the WST-8/PMS-based CCK-8 assay [24, 25, 27]. The reaction proceeds in two coupled steps: (i) any NADPH remaining after the GPx/GR-coupled reaction has run to completion reduces 1-mPMS, itself becoming oxidized to NADP^+^, while 1-mPMS is reduced to its leuco (reduced) form, 1-mPMS(red). (ii) 1-mPMS(red) then rapidly and non-enzymatically reduces the tetrazolium ring of WST-8, cleaving it to the water-soluble, orange formazan dye (λmax ≈ 460 nm) and regenerating oxidized 1-mPMS, which re-enters the cycle.

Because step (ii) is fast relative to step (i), 1-mPMS shuttles electrons from residual NADPH to WST-8 without itself becoming rate-limiting, so the amount of formazan formed within the fixed 10-minute incubation is directly proportional to the concentration of residual NADPH remaining at the end of the coupled enzymatic reaction. Since residual NADPH is inversely related to GPx activity, higher GPx activity drives more NADPH consumption through the coupled GR reaction, leaving less NADPH available to reduce WST-8; the resulting absorbance at 460 nm is inversely proportional to GPx activity.

**Figure 3:**
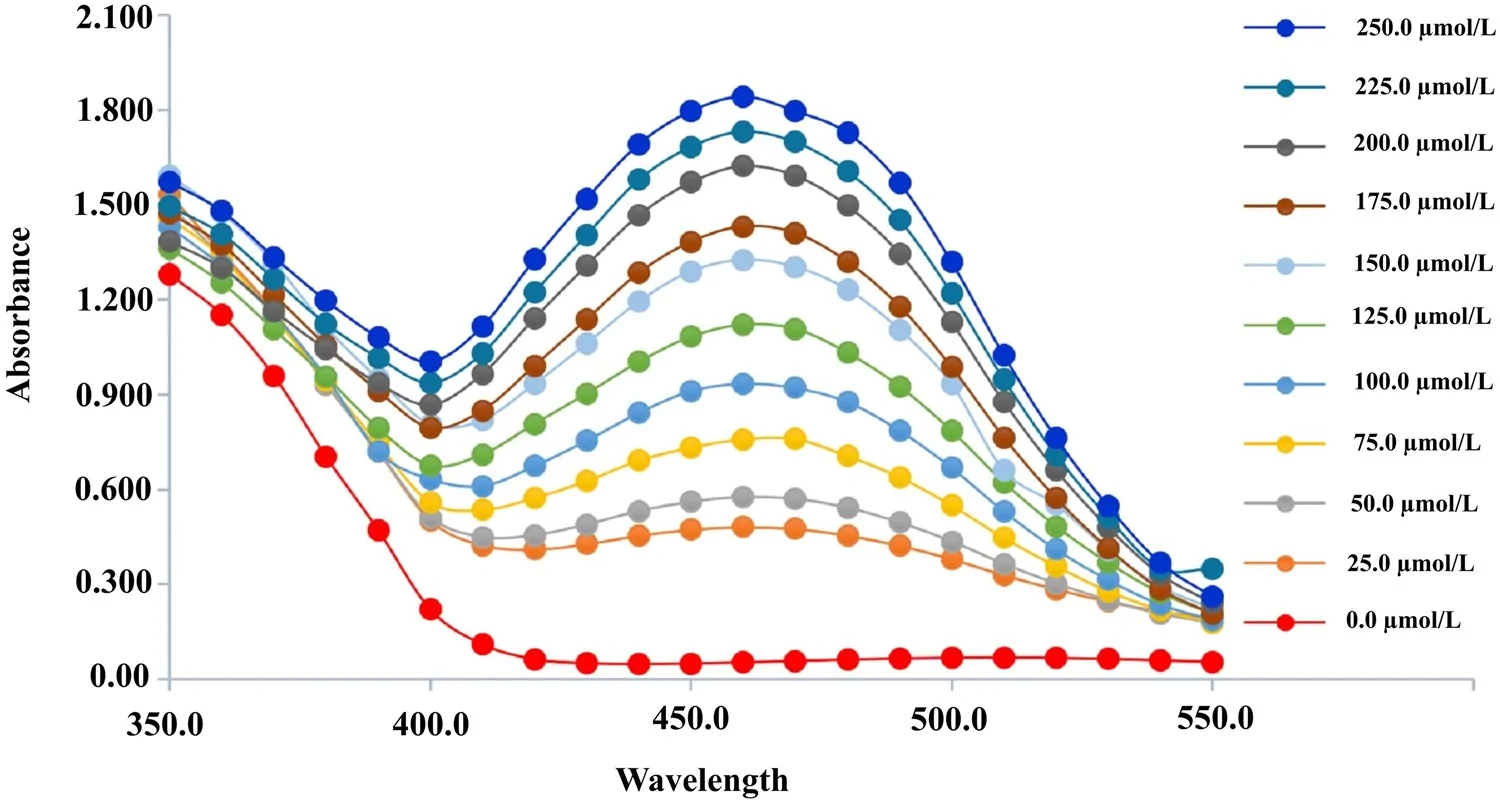
Absorbance spectra (350–550 nm) of the WST-8/1-mPMS reaction product recorded at NADPH concentrations of 25–250 µmol/l. Absorbance at 460 nm decreases as NADPH concentration increases, reflecting the inverse relationship between residual NADPH and GPx activity.

Absorbance spectra recorded between 350 and 550 nm confirmed the expected relationship between residual NADPH and the WST-8 signal: as the NADPH concentration in the reaction mixture increased from 25 to 250 µmol/l, the absorbance at 460 nm decreased progressively (Fig. 3), consistent with the inverse relationship between residual NADPH and GPx activity that underlies the assay.

### Linearity and analytical sensitivity

The GPx–WST8 assay exhibited excellent linearity over a wide NADPH concentration range (0.5–250 µM), with a Pearson correlation coefficient of *r = *0.99 (Fig. 1). This strong linear relationship confirms the validity of the calibration curve used to convert absorbance values into residual NADPH concentrations throughout the assay’s entire working range.

The assay also demonstrated high analytical sensitivity, with a limit of detection (LOD) of 1 U/l and a limit of quantification (LOQ) of 3 U/l. These low detection limits enable accurate, reliable quantification of GPx activity in samples with very low enzyme levels, thereby extending the method’s applicability to a wide range of biological specimens and research settings.

### Control of matrix effects

Endogenous NADH, NADPH, and other reducing substances present in biological specimens may interfere with indirect GPx assays by contributing to background absorbance. To minimize these matrix effects, a parallel control tube containing all assay components except GPx was included for each sample.

The absorbance measured in the control tube represented the background signal arising from endogenous chromogens and other matrix constituents, whereas the absorbance of the corresponding test tube comprised both the background signal and the GPx-dependent response [28]. Consequently, subtracting the control absorbance from the test absorbance effectively eliminated matrix-derived interference and isolated the net GPx-specific signal. This correction ensured accurate and reliable quantification of GPx activity across a wide range of biological samples with varying endogenous matrix compositions.

### Accuracy, selectivity, and recovery

The accuracy and selectivity of the GPx–WST8 assay were assessed by determining the recovery of a known GPx activity (250 U/l) following its addition to phosphate buffer containing representative physiological interferents, including carbohydrates, amino acids, and proteins, at concentrations designed to simulate complex biological matrices (Table 2).

**Table 2:** Recovery of GPx activity in the presence of carbohydrate, amino acid, and protein interferents, determined using the GPx-WST8 assay.

Flask no.	Contents	Added GPx (U/l)	Found GPx (U/l)	Recovery (%)
1	Phosphate buffer (pH 7.2; 100 mM) – control, no interferent	250	250	—
2	Fructose, xylose, and sucrose (2 mM each)	250	244	97.6
3	Histidine, cysteine, methionine, and threonine (2 mM each)	250	248	99.2
4	0.5% Bovine serum albumin (BSA) + 0.5% casein	250	255	102.0

The measured GPx recoveries ranged from 97.6% to 102.0%, demonstrating excellent assay accuracy and indicating that the tested carbohydrates, amino acids, and proteins did not produce significant interference under the experimental conditions. These findings confirm the high selectivity of the GPx–WST8 assay for GPx activity, even in the presence of common endogenous matrix constituents.

The ability to accurately quantify GPx without significant matrix interference minimizes the need for extensive sample pretreatment or purification. Consequently, the assay reduces sample processing time, reagent consumption, and potential analyte loss, making it particularly suitable for routine analysis and research-based applications.

### Method comparison and validation

The analytical performance of the GPx–WST8 assay was validated by comparison with two established reference methods, GPx–DTNB and GPx–UV, using Bland–Altman analysis and Passing–Bablok regression (GraphPad Prism, GraphPad Software, San Diego, CA, USA). Paired measurements were obtained from enzyme samples spanning the assay’s analytical range (2–250 U/l), generated by serial dilution to ensure comprehensive evaluation across low, intermediate, and high GPx activities.

**Figure 4:**
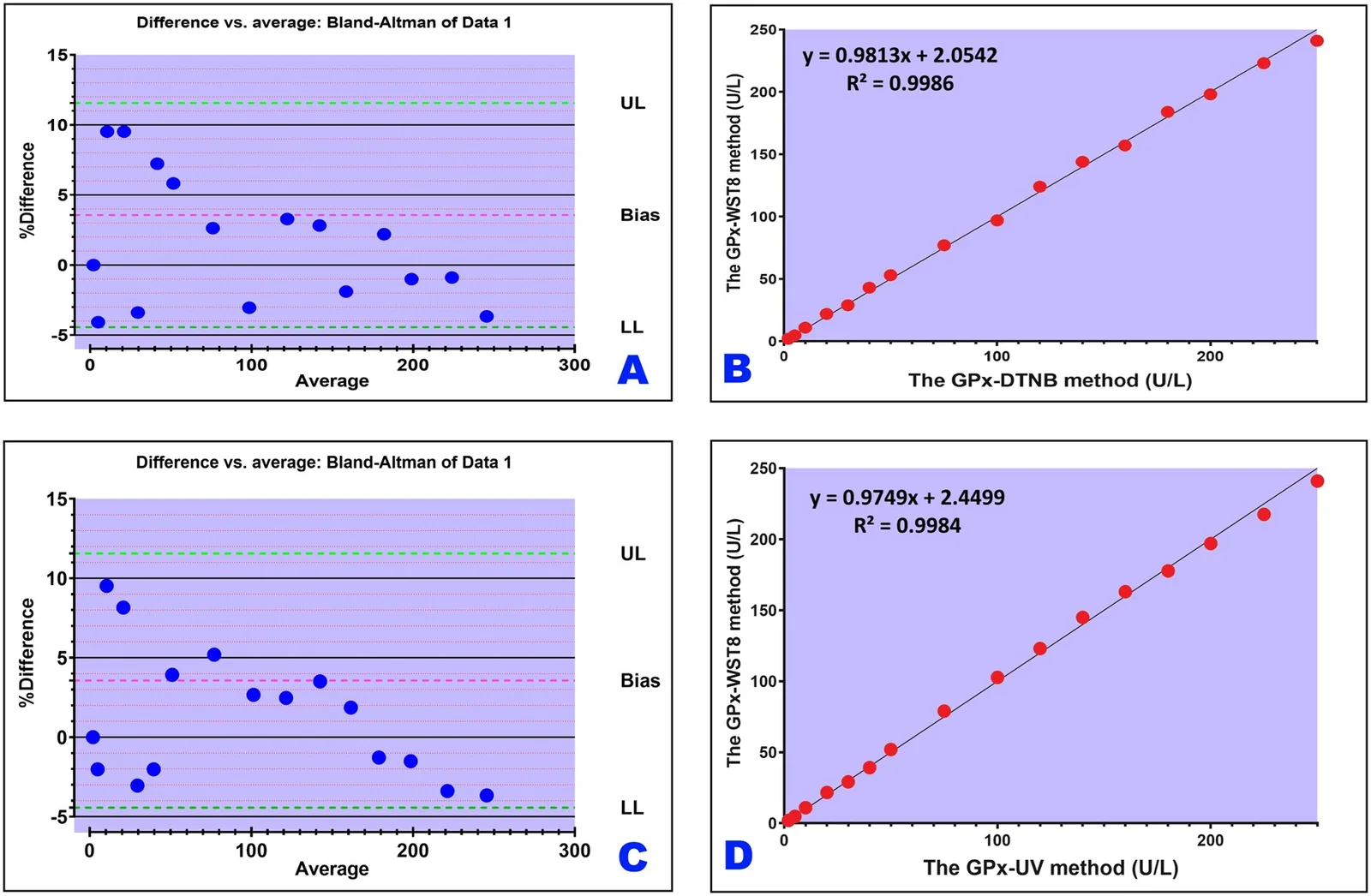
Method-comparison analyses for the GPx-WST8 assay. (A) Bland-Altman plot, GPx-WST8 versus GPx-DTNB. (B) Passing-Bablok regression, GPx-WST8 versus GPx-DTNB. (C) Bland-Altman plot, GPx-WST8 versus GPx-UV. (D) Passing-Bablok regression, GPx-WST8 versus GPx-UV.

Bland–Altman analysis demonstrated excellent agreement between the GPx–WST8 assay and both reference methods. Comparisons with the GPx–DTNB assay (Fig. 4A) and the GPx–UV assay (Fig. 4C) revealed minimal mean bias and narrow limits of agreement, indicating the absence of clinically or analytically significant systematic error over the tested concentration range.

The results were further supported by Passing–Bablok regression analysis, which demonstrated an excellent linear relationship between the GPx–WST8 assay and the GPx–DTNB method (*r = *0.99; *y = *0.9813*x* + 2.0542; Fig. 4B) as well as the GPx–UV method (*r = *0.99; *y = *0.9749*x* + 2.4499; Fig. 4D). In both comparisons, the regression slopes were close to the ideal value of 1.0, while the intercepts were small, indicating negligible proportional and constant bias. Collectively, these findings demonstrate that the GPx–WST8 assay yields results highly consistent with those obtained using established reference methods, supporting its accuracy, reliability, and suitability for routine determination of GPx activity in biological samples.

### Application to biological samples

The applicability of the GPx–WST8 assay to complex biological matrices was evaluated by measuring GPx activity in rat serum, erythrocytes, kidney, and liver homogenates using three peroxide substrates, H_2_O_2_, tBuOOH, and CuOOH. The results were compared with those obtained using the established GPx–UV reference method (Table 3).

**Table 3:** Comparative analysis of GPx activity in rat tissue homogenates using the GPx-WST8 method and the GPx-UV and GPx-DRNB reference methods.

Sample	Peroxide substrate	GPx-WST8 method	GPx-UV method
		Mean ± SD	Mean ± SD
Serum (U/l)	H_2_O_2_	381 ± 7	377 ± 4
	tBuOOH	351 ± 4	357 ± 3
	CuOOH	355 ± 3	363 ± 5
Erythrocytes (U/g Hb)	H_2_O_2_	191 ± 4	196 ± 4
	tBuOOH	172 ± 2	176 ± 3
	CuOOH	188 ± 3	193 ± 4
Kidney (U/mg protein)	H_2_O_2_	141 ± 2	141 ± 3
	tBuOOH	125 ± 1	122 ± 2
	CuOOH	131 ± 2	133 ± 3
Liver (U/mg protein)	H_2_O_2_	288 ± 3	282 ± 4
	tBuOOH	278 ± 3	271 ± 5
	CuOOH	288 ± 3	283 ± 4

Across all biological matrices and peroxide substrates, the GPx–WST8 assay produced GPx activity measurements that closely matched those obtained by the GPx–UV method. The slight differences observed between the two methods were well within the measurement standard deviations, demonstrating excellent analytical agreement and supporting the accuracy of the proposed assay in biologically relevant samples. For all 12 sample/substrate combinations, GPx-DTNB values aligned closely with GPx-WST8, with absolute differences ranging from 0% to 3.1% (average 1.2%). In every instance, the discrepancy between methods was contained within the standard deviation of at least one measurement. These findings confirm that the GPx-WST8 assay is consistent with the GPx-DTNB reference method in complex biological matrices, thereby extending the established concordance with the GPx-UV method.

As expected, GPx activity varied among tissues and peroxide substrates, reflecting the differential expression of GPx isoenzymes and their distinct substrate specificities. Notably, liver homogenates exhibited the highest GPx activities, consistent with the liver’s well-established role as a major site of antioxidant defense and peroxide detoxification. The close agreement between the GPx–WST8 and GPx–UV methods across all sample types and substrates demonstrates the robustness, accuracy, and broad applicability of the GPx–WST8 assay for measuring GPx activity in diverse biological specimens.

## Discussion

Reliable quantification of GPx activity remains central to oxidative stress research and to the clinical assessment of antioxidant status, yet each established assay format has practical limitations. The DTNB method is relatively insensitive and requires acid-mediated termination and protein precipitation, complicating its adaptation to microplate formats [15]. The coupled GR-NADPH/UV method of Paglia and Valentine [16] allows continuous kinetic monitoring but is compromised by strong UV background absorbance from co-extracted proteins and nucleic acids at 340 nm, by potential NADPH-mediated inhibition of GPx, and by the need for tightly controlled reaction temperatures and UV-capable spectrophotometry [17]. The CUPRAC method is susceptible to interference from endogenous catalase [18, 19], fluorometric assays require derivatization steps and specialized instrumentation [20], and the more recently described Tiron-based protocol, while accurate, depends on freshly prepared reagents and strict pH control [21]. The GPx-WST8 assay described here was designed to retain the sensitivity of these approaches while removing their main sources of interference and operational complexity.

The wide linear range (0.5–250 µM) and low detection limits (LOD 1 U/l; LOQ 3 U/l) obtained in the present study indicate that an endpoint colorimetric readout at 460 nm can match the sensitivity of the traditional 340 nm kinetic method without incurring its principal drawback: because the WST-8 formazan product absorbs well outside the UV range, the assay is largely free of the background absorbance from proteins and nucleic acids that limits the sensitivity of the GR-NADPH/UV method in complex tissue homogenates [17].

The recovery data obtained in the presence of sugars, amino acids, and proteins (97.6%–102.0%) show that the simple background-subtraction strategy used here, running a parallel control tube for every sample, is sufficient to control matrix interference without the additional termination or precipitation steps required by the DTNB- and Tiron-based methods [15, 21]. Combined with the assay’s sensitivity, this selectivity translates into practical benefits for high-throughput use: fewer sample-preparation steps, lower reagent consumption, and a reduced risk of analyte loss during workup.

Perhaps most importantly, the strong agreement between the GPx-WST8 method and both the GPx-DTNB and GPx-UV reference assays, evidenced by narrow Bland-Altman limits of agreement and Passing-Bablok regression slopes close to unity with minimal intercepts, demonstrates that the simplicity of the new assay does not come at the expense of accuracy. Achieving equivalence with the GR-NADPH/UV method, which remains the most widely used indirect assay for GPx activity [16], is a meaningful benchmark for any proposed alternative. This concordance extended beyond simple buffer systems: GPx-WST8 values obtained in serum, erythrocyte, kidney, and liver homogenates, across three chemically distinct peroxide substrates, closely tracked those obtained by the GPx-UV method, supporting the applicability of the assay to real, complex biological matrices rather than to spiked buffer systems alone.

Beyond its analytical performance, the GPx-WST8 assay offers several practical advantages relevant to routine use. The commercial WST-8/1-mPMS detection reagent is stable and requires no fresh daily preparation, in contrast to the GSH and NADPH stocks required by the assay itself and by several reference methods, which must be prepared fresh to avoid oxidative loss of activity. The assay is performed in a standard microplate format using visible-range spectrophotometry, avoiding the UV-capable instrumentation and precise thermal control required by the GR-NADPH/UV method [17], and its reagents are less hazardous to handle than some used in traditional GPx assays. Together, these features are consistent with the need identified in the Introduction section for a sensitive, cost-effective, and interference-resistant GPx assay suited to laboratories with limited access to specialized instrumentation.

This study also has limitations that warrant consideration. Like the GPx-UV method against which it was validated, the GPx-WST8 assay is an indirect, coupled-enzyme method, so its accuracy depends on adequate GR activity and the freshness of the GSH and NADPH reagents. To date, validation has focused on spiked buffer systems and animal tissue homogenates; broader validation in human clinical samples across a wider range of physiological and pathological states, together with multi-center reproducibility studies, would further support the assay’s adoption as a routine diagnostic tool.

## Conclusion

This study describes a WST-8-based colorimetric endpoint assay for GPx activity that combines high sensitivity (LOD, 1 U/l; LOQ, 3 U/l), a wide linear range (0.5–250 µM), and strong resistance to interference from sugars, amino acids, and proteins (recovery, 97.6%–102.0%) with excellent agreement against the established GPx-DTNB and GPx-UV reference methods (*r = *0.99; regression slopes close to unity). Successful application to serum, erythrocyte, kidney, and liver homogenates across multiple peroxide substrates further confirms the assay’s suitability for complex biological specimens.

Taken together with its operational simplicity, reagent stability, low cost, and compatibility with standard microplate spectrophotometry, the GPx-WST8 assay offers a practical and broadly accessible alternative to existing GPx methods for both oxidative stress research and routine diagnostic use, including in laboratories with limited access to specialized UV instrumentation. Further validation in human clinical cohorts is warranted to support its translation into routine diagnostic practice.

## Data Availability

The authors declare that all data supporting this study’s findings are included in the article. Further data are available from the corresponding author upon request.
